# Physical Carboxymethylscleroglucan/Calcium Ion Hydrogels as Modified Drug Delivery Systems in Topical Formulations

**DOI:** 10.3390/molecules14082684

**Published:** 2009-07-24

**Authors:** Federica Corrente, Pietro Matricardi, Patrizia Paolicelli, Beatrice Tita, Federica Vitali, Maria Antonietta Casadei

**Affiliations:** 1 Dipartimento di Chimica e Tecnologie del Farmaco, “Sapienza” Università di Roma, Piazzale Aldo Moro 5, 00185, Rome, Italy; 2 Dipartimento di Fisiologia e Farmacologia, “Sapienza” Università di Roma, Piazzale Aldo Moro 5, 00185, Rome, Italy

**Keywords:** carboxymethyl scleroglucan, physical hydrogels, controlled release, rheology, mucoadhesiveness, primary skin irritation

## Abstract

A carboxymethyl derivative of scleroglucan (Scl-CM) with a 65±5% carboxylic group degree of derivatization (DD) was recently synthesized and characterized. Aqueous solutions of the polymer underwent to a sharp transition toward a gel like behaviour in the presence of divalent ions such as Ca^+2^. Physical hydrogels with different Scl-CM/Ca^+2^ ratios were prepared and characterized for their rheological behaviour. Their potential as drug delivery systems was also evaluated. To this end three non steroidal anti-inflammatory drugs (NSAIDs) were loaded into the hydrogels obtained with 2% w/v solution of Scl-CM and 0.05 and 0.1 M CaCl_2_. The release rate of the drugs was critically related to the salt concentration. By an appropriate combination of the hydrogels prepared using different amounts of salt, it was possible to obtain a system able to release diclofenac with zero-order kinetics. Primary skin irritation tests showed a good biocompatibility of the new polymer, as well as of its hydrogels. These results suggest a potential of the new hydrogels for the development of modified delivery systems in topical formulations.

## 1. Introduction

Polysaccharides and their derivatives represent a class of polymers widely used in pharmaceutical formulations. In particular they find employment as starting materials for the preparation of hydrogels, three-dimensional networks able to retain large amounts of water or biological fluids. As a consequence, these systems show a consistence very similar to natural living tissues that allows their use for several biomedical applications [1,2,3,4]. Among polysaccharides, scleroglucan (Scl) has been proposed for the preparation of effective modified drug delivery systems, a topic that has recently been reviewed [5]. Scl is a water-soluble extra-cellular polysaccharide produced by fungi of the genus *Sclerotium*. It consists of a backbone of (1→3)-β-linked glucose residues substituted with single (1→6)-β-D-glucopyranosyl residues on every third backbone unit. Scl adopts in water a triple helix conformation that can be destroyed by dimethylsulfoxide and in solutions at pH ≥12.5 [6,7]. The controlled oxidation of Scl produces the opening of the glucose in the side chain with the formation of the aldehyde derivative [8] that can be further oxidized to the corresponding carboxylic acid (Sclerox) [9]. Sclerox is able to form chemical gels by reaction with 1,6-dibromohexane [10] or physical gels in the presence of calcium ions [5], both proposed as drug delivery systems.

Recently our group synthesized a carboxymethyl derivative (Scl-CM) of scleroglucan able to form physical hydrogels with divalent ions, such as Ca^+2^ [11]. Systems with different polymer/salt ratio were prepared and evaluated as controlled delivery systems of drugs, using acyclovir as a model. The release rate of the drug critically depended on the concentration of the salt and the molar ratio carboxylated repetitive units of the polymer/Ca^2+^.

Starting from these preliminary results, we wish now to report further studies performed on Scl-CM and its hydrogels, aimed at investigating their potential as drug delivery systems. First of all we planned to verify if the new systems could be used for the development of topical formulations containing drugs of different nature. To this end, some non steroidal anti-inflammatory drugs (NSAIDs) were chosen because of the several and well-known adverse effects associated to their systemic use, especially towards the gastrointestinal tract [12]. In order to avoid these side effects and prolong their activity, NSAIDs are often formulated as modified delivery systems in topical preparations. Three among the most used NSAIDs (ibuprofen, ketoprofen and diclofenac) were loaded into the hydrogels obtained from 2% w/v aqueous solutions of Scl-CM and two different concentration of CaCl_2_ (0.05 and 0.1 M), and the corresponding release profiles were investigated. Moreover the possibility of developing a system able to release a drug according to zero-order kinetics was also evaluated. To this end diclofenac was selected, as it is available on the market in several topical preparations, such as gels, creams and patches. The rheological behaviour of the hydrogels containing or not diclofenac was investigated. Finally primary skin irritation studies were performed on rabbits.

## 2. Results and Discussion

### 2.1. Synthesis of Scl-CM

**Figure 1:**
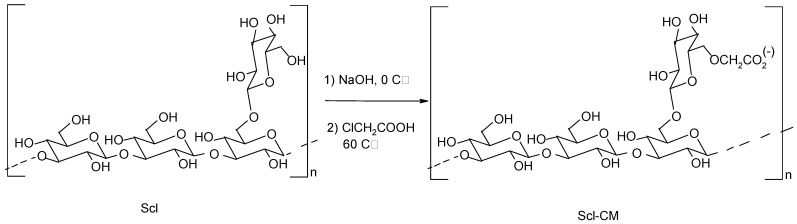
Scheme of Scl-CM synthesis.

Scleroglucan is a neutral homopolysaccharide that can be modified by reaction with choloroacetic acid in basic media as previously reported [11,13]. In this way the neutral polysaccharide can be converted into a negatively charged polymer (Scl-CM). The synthesis scheme is shown in Figure 1.

**Figure 2:**
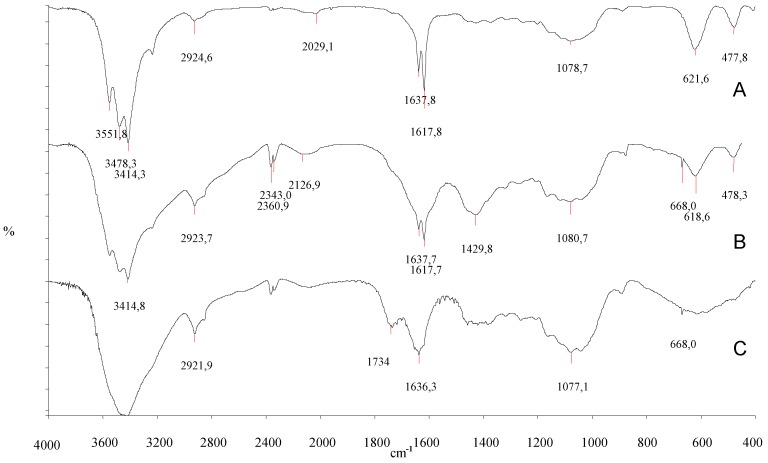
FT-IR spectra of Scl (**A**), Scl-CM before (**B**) and after (**C**) acidification.

The obtained polymer was characterized by FT-IR analysis. The spectra of the starting scleroglucan and of Scl-CM before and after the treatment on an ion-exchange resin are shown in Figure 2.

In the Scl-CM sample (**B**) a more intense band than in the Scl spectrum (**A**) can be observed at 1,618 cm^-1^, probably related to the asymmetric stretching vibration of the carboxylate group. The acidification of the derivative produces the disappearance of this peak and the appearance of a new peak at 1,734 cm^-1^, probably due to the stretching vibration of the C=O in the undissociated acid.

### 2.2. In vitro evaluation of the mucoadhesive properties of Scl-CM

**Figure 3:**
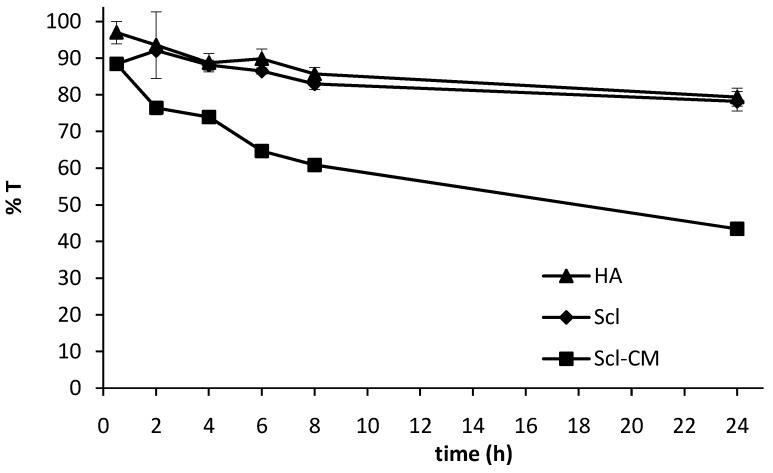
Transmittance % values at 500 nm of mucin dispersions in the presence of Scl (♦), Scl-CM (■) and hyaluronic acid, HA (▲) chosen as a positive control, as a function of the time.

Mucoadhesion is a topic of current interest in the design of drug delivery systems. Mucoadhesive drug delivery systems prolong the residence time of the dosage form at the site of application and facilitate an intimate contact of the dosage form with the underlying absorption surface, thus contributing to an improved therapeutic performance of the drug. Therefore, the mucoadhesive properties of the new polymer were investigated. The ability of Scl and Scl-CM to interact with mucin was evaluated by a turbidimetric method and compared with hyaluronic acid, used as a positive control. Since Scl and Scl-CM do not produce clear solutions, neither in distilled water nor in phosphate buffer, the polymers were centrifuged before the assays, in order to remove eventual macro-aggregates that would interfere with the turbidimetric evaluation. Then the polymers were added to a 1% w/v mucin suspension and after 30 min, 2, 4, 6, 8 and 24 hours of incubation, the transmittance values of the polymer-mucin mixtures were determined at 500 nm. The results are reported in Figure 3.

The greater the interaction between the polymer and mucin is, the lower is the transmittance value of the tested sample (greater UV-Vis scattering), due to the formation of macro-aggregates [14,15]. Therefore it can be observed that under the adopted experimental conditions, Scl presents the same ability to interact with mucin as hyaluronic acid, whereas the insertion of carboxylic groups on its backbone caused a marked increase in the mucoadhesive properties of the polymer. In fact, lower transmittance values were measured for the Scl-CM/mucin mixture than for Scl and HA/mucin ones, probably because the presence of the carboxylic groups increases the hydrogen bonds responsible for the polymer-mucin interactions.

### 2.3. Release studies from physical hydrogels of Scl-CM 

Physical hydrogels of Scl-CM were prepared taking into account a previous report [11], where preliminary studies were performed in order to find the experimental conditions for the hydrogels formation. In that work two polymer concentrations (1 and 2% w/v) and different amounts of salt were investigated. On the basis of these preliminary studies, the 2% w/v concentration was now chosen for further investigation, because it allowed us to explore a wider range of CaCl_2_ concentrations. In particular hydrogels with 0.05, 0.10, 0.25 and 0.50 M of CaCl_2_ and loaded with 0.25% w/v acyclovir can be prepared in these conditions. The release rate of the drug from the four Scl-CM hydrogels (C_p_2-C_s_0.50; C_p_2-C_s_0.25; C_p_2-C_s_0.10; C_p_2-C_s_0.05) was studied in phosphate buffer (PB) at pH=7.4 and the results reported in Table 1.

**Figure 4:**
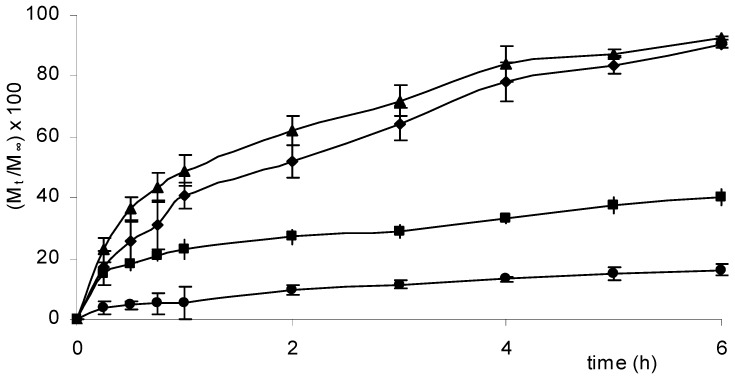
Release profiles [(M_t_/M_∞_)×100] of acyclovir from hydrogels Cp2-Cs0.05 (▲), Cp2-Cs0.10 (♦), Cp2-Cs0.25 (■) and Cp2-Cs0.50 (●), maintained in PB solution (pH=7.4) at 37.0 ± 0.1°C for 6 hours.

Furthermore for an easier comparison, the release profiles of acyclovir from the four systems are reported in Figure 4. 

As it can be observed, the release rate of acyclovir depends critically on the amount of salt used to prepare the matrices – the higher the CaCl_2_ concentration, the slower the release. The matrices having the higher salt concentrations were unable to release sufficient amounts of drug either in 6 h, the usual permanence time of topical formulations, nor in 24 h, the maximum application time of transdermal patches. On the contrary, a quite complete release was observed from hydrogels prepared using salt concentrations lower than 0.1 M. As a consequence, systems with 0.05 and 0.1 M CaCl_2_ were considered for further investigation because they allowed the quantitative release of the drug in 24 h.

**Figure 5:**
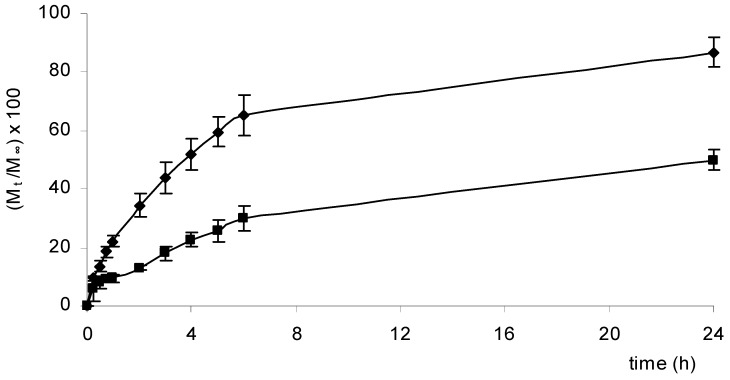
Release profiles [(M_t_/M_∞_)×100] of ketoprofen from hydrogels Cp2-Cs0.05 (♦) and Cp2-Cs0.10 (■) maintained in PB solution (pH=7.4) at 37.0 ± 0.1 °C for 24 hours.

**Figure 6:**
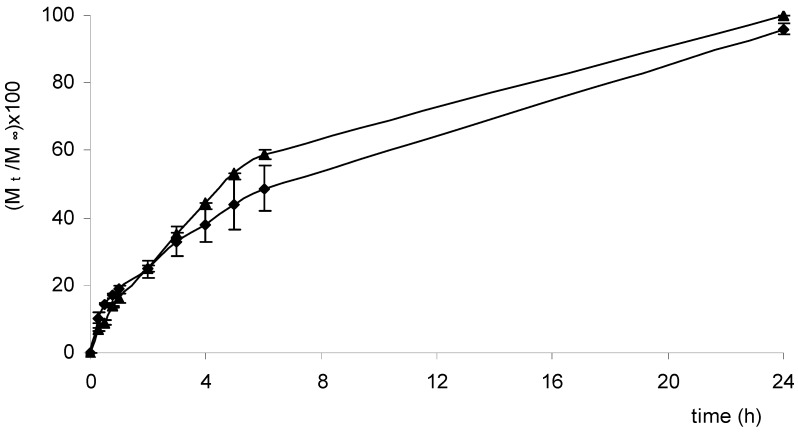
Release profiles [(M_t_/M_∞_)×100] of ibuprofen from hydrogels Cp2-Cs0.05 (▲) and Cp2-Cs0.10 (♦) maintained in PB solution (pH=7.4) at 37.0 ± 0.1 °C for 24 hours.

**Figure 7:**
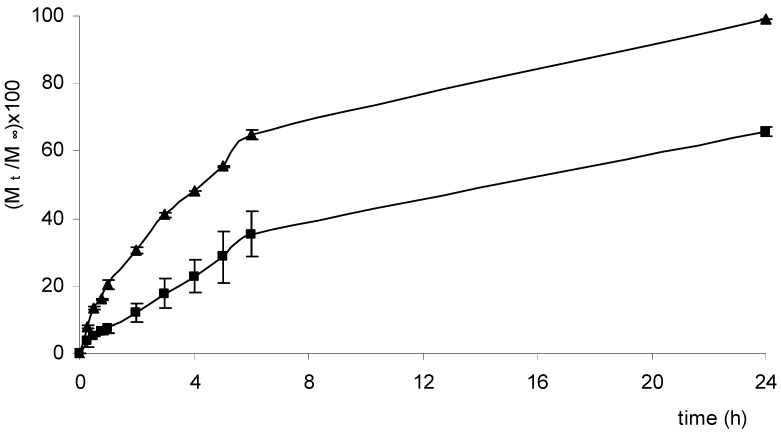
Release profiles [(M_t_/M_∞_)×100] of diclofenac from hydrogels Cp2-Cs0.05 (▲) and Cp2-Cs0.10 (■) maintained in PB solution (pH=7.4) at 37.0 ± 0.1 °C for 24 hours.

Three anti-inflammatory drugs (NSAIDs) – ketoprofen, ibuprofen and diclofenac – among the most widely used for oral as well as for topical administration, were loaded into the two selected matrices (Cp2-Cs0.1 and Cp2-Cs0.05). As stated before, NSAIDs are often formulated for topical applications, for example in gels and patches, in order to avoid their well-known adverse effects associated with their systemic use, especially towards the gastrointestinal tract. In fact a high incidence of gastric and duodenal ulcerations has been reported for their oral use [12]. The release profiles of ketoprofen, ibuprofen and diclofenac from Scl-CM based hydrogel are reported in Figure 5, Figure 6 and Figure 7.

As already observed for acyclovir, even for these drugs the higher the salt concentration is, the slower is the release rate. Differences in the release rates of ibuprofen respect to ketoprofen and diclofenac may probably be related to differences in structure and dimensions of the molecules. Experiments performed in medium at pH=5.5, miming skin conditions, showed a release rate only slightly slower than that obtained in PB solution, as already reported for acyclovir [11].

### 2.4. Release studies of diclofenac from a double-layered system

**Figure 8:**
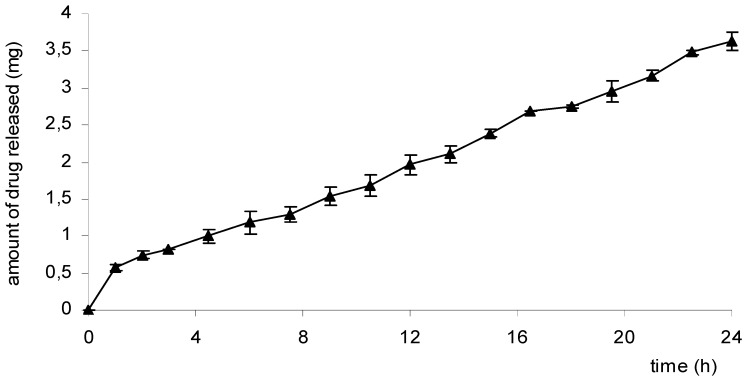
Release profile of diclofenac from the system at double-layer maintained in PB solution (pH=7.4) at 37.0 ± 0.1 °C for 24 hours.

As observed before, the release rate of a drug from Scl-CM/CaCl_2_ hydrogels could be easily modified changing the salt concentration. It is well known that systems able to release a drug according to zero-order kinetics could be very useful for long-term medications, reducing the number of daily applications. Therefore, we investigated the possibility of developing a sustained delivery system based on Scl-CM/CaCl_2_ hydrogels by combining hydrogels at different salt concentration. To this aim a double-layered sample constituted by an outer layer of a looser hydrogel (Cp2-Cs0.05) and an inside layer of a stronger hydrogel (Cp2-Cs0.10) both loaded with diclofenac sodium salt (0.5% w/v), was prepared. The release profile of diclofenac from this system is reported in Figure 8 as the amount of the released drug as a function of the time.

A burst effect is evident at the first hour, soon followed by a zero-order release kinetic. It is likely that when the outer layer becomes depleted in the drug, it is replaced by other drug coming from the deeper layer with a slower rate, so the system is able to release a constant amount of diclofenac for at least 24 hours after the application. As a consequence the combination of layers of hydrogels at different salt concentration could constitute a very easy method to build a system able to release the drug at the rate and in the amount requested by the therapeutic necessities.

### 2.5. Rheological measurements 

The rheology of a topical formulation is among the most important properties to be measured, since it influences the ability to spread over the skin and mucosae and it can also affect the performance of the formulation. Therefore the rheological behaviour of Scl-CM based hydrogels was evaluated. To this end aliquots of CaCl_2_ water solutions were added to samples of 2% w/v Scl-CM water solutions in order to obtain hydrogels with salt concentrations of 0.05, 0.1, 0.25 and 0.5 M; the thus obtained hydrogels were submitted to rheological investigation.

**Figure 9:**
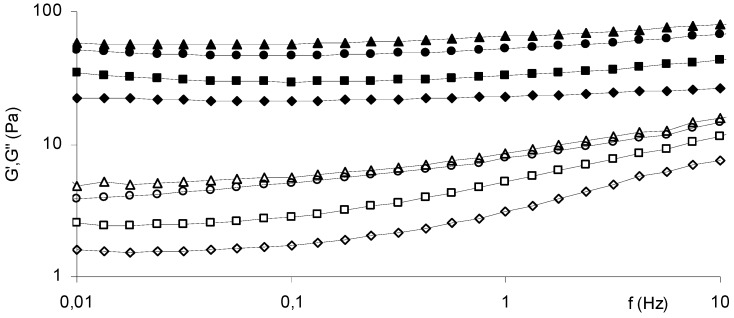
Mechanical spectra of the hydrogels Cp2-Cs0.05 (G’♦, G’’ ◊), Cp2-Cs0.10 (■ G’, □ G’’), Cp2-Cs0.25 (● G’, ○ G’’) and Cp2-Cs0.50 (▲G’, ∆ G’’) at 25.0 ± 0.2 °C.

The rheological properties of the hydrogels obtained with 2% w/v Scl-CM were not influenced by the temperature, so all the experiments were performed at room temperature [16]. In Figure 9 the mechanical spectra, i.e. the elastic (G’) and the loss (G’’) moduli profile as a function of the mechanical shear deformation applied to the samples at different frequencies, are reported for each hydrogel prepared. The quite parallel profile of the G’ and G’’ curves for each concentration is a typical behaviour of a hydrogel. As shown in the Figure 9, an increase of the salt concentration results in an increase of the G’ and the G’’. In detail, the G’ value, that is a measure of the strength of the gel, increases as the CaCl_2_ increases from 0.05M to 0.25M. The sample Cp2-Cs0.50, i.e. with the highest concentration of salt, shows the G’ value almost identical to Cp2-Cs0.25 one, evidencing that at 0.25 M the maximum interaction between polymer and salt is reached. 

**Figure 10:**
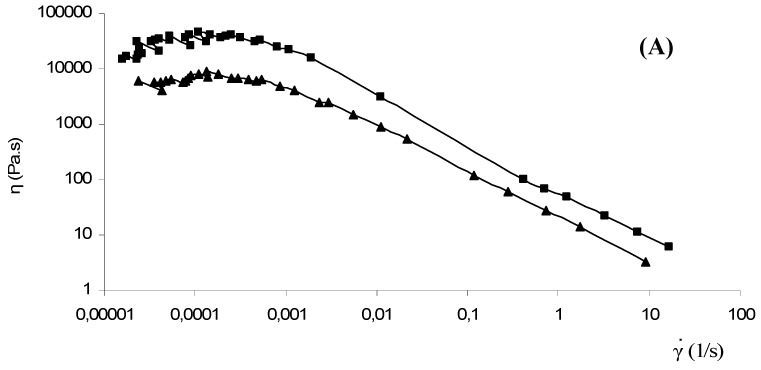

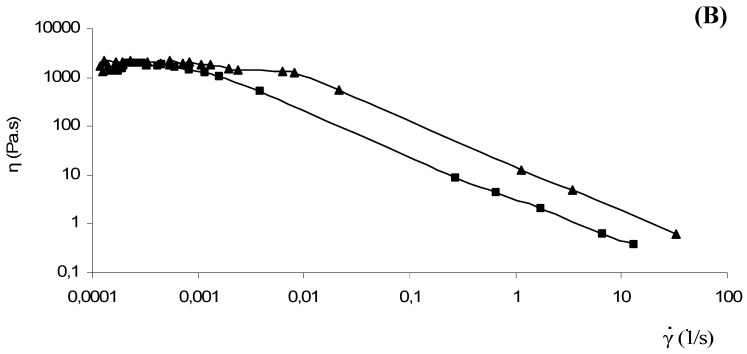
Flow curves of the hydrogels unloaded (**A**) and loaded (**B**) with diclofenac sodium salt: ■ Cp2-Cs0.05 and ▲Cp2-Cs0.1 at 25 ± 0.2 °C.

The viscosity (η) as a function of the shear rate (γ**^.^**) for each hydrogel was also recorded in order to assess the ability of the systems prepared to be spread for topical applications. The physical hydrogels are able to flow and a pseudoplastic behaviour was observed. In Figure 10 (A) the flow curves of two of the hydrogels prepared (Cp2-Cs0.05 and Cp2-Cs0.1) are reported. At very low shear rate the values of the shear viscosity are above 10^3^ Pa.s, whereas above 10 s^-1^, in the range of shear rates usually adopted during topical applications, the shear viscosity drops at values lower than 10^1^ Pa.s.

In Figure 10 (B) the same experiments were carried out on hydrogel loaded with diclofenac. The behaviour appear to be quite similar; at low shear rate the shear viscosity of the hydrogel was affected by the presence of the drug with respect to the unloaded hydrogel.

The effect of the drug loading on the shear rate viscosity of the hydrogels, with a reduction of the absolute values with respect to the unloaded hydrogels, can be ascribed to the disturbing effect on the network formation exerted by the host molecules. This negative interacton between the drug molecules and the network structure is more evident in the low shear rate regime region, because the measurements performed applying small deformations, preserve the overall loaded hydrogel structure. In this conditions the more structured hydrogel, i.e. the one with the higher salt concentration (G’ increases as the CaCl_2_ concentration is increased, see Figure 9) experiences deeper modification. From a physical point of view, this behaviour could be related to a limitation of the self-structuring ability of the Scl-CM/CaCl_2_ system due to the host molecules, thus levelling the low shear viscosity among the systems.

On the other hand, at higher shear rate conditions, the networks are in a large deformations regime; in this case, in the framework above depicted, the networks are continuously modified and the onset of the pseudoplasticity is more evident for the system with the higher CaCl_2_ concentration because of a reduced mobility of the Scl-CM chains. The overall results suggest that the spread-ability and the adherence of the materials is suitable for the application and performance on the skin and mucosal surfaces [17].

### 2.6. Primary skin irritation experiments 

**Figure 11:**
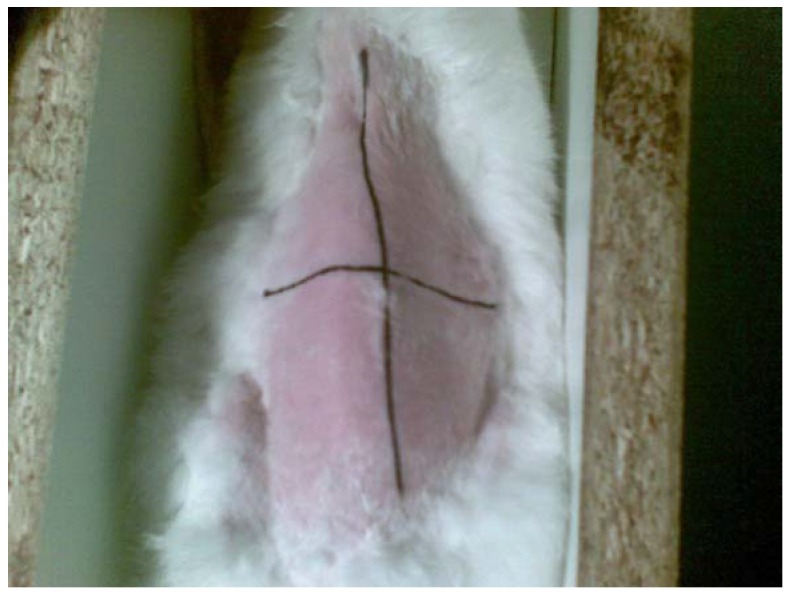
The back of the animals clipped and divided into four sites, after 24h from the application of Scl, Scl-CM and Scl-CM/CaCl_2_.

Primary skin irritation experiments were carried out in order to verify a possible use of the new polymer and of its physical hydrogels in topical formulations. The tests were performed on rabbits. Individual skin irritation score results, calculated as described in the Experimental part, were always equal to 0. In the animals erythema or edema were absent after 24, 48 and 72 h of application of the three samples (Scl, Scl-CM and Scl-CM/Ca^2+^) as well as in the control (Figure 11). According to our data, the Primary Skin Irritation Index (PII) of the tested materials was negligible, showing the absence of skin irritation, so that they could be useful as modified drug delivery system in topical formulations.

## 3. Experimental Section

### 3.1. Materials

All used reagents were of analytical grade. Scleroglucan with Mw=1.4 x 10^6^ as evaluated by viscosimetric measurements in 0.01 M NaOH, was provided by Carbomer. Dimethylsulfoxide (DMSO), CaCl_2_ x 2H_2_O, ibuprofen sodium salt and chloroacetic acid were purchased from Fluka (Switzerland). Ketoprofen, diclofenac sodium salt, acetonitrile, D_2_O, DMSO-d6 and DOWEX 50WX4-50 ion-exchange resin were purchased from Aldrich (England). Acyclovir was kindly provided as a gift by Recordati (Italy).

### 3.2. Synthesis of carboxymethyl scleroglucan (Scl-CM)

Scleroglucan (1.0 g) was dissolved in water (20 mL) at 90 °C and to the solution, transferred in ice bath, NaOH (14.3 g) was added. After complete dissolution, a solution of chloroacetic acid (5.0 g) in water (25 mL) was added dropwise. The mixture was maintained under stirring at 60 °C for 24 h. After neutralization with glacial acetic acid, the solution was submitted to exhaustive dialysis. In order to obtain all the carboxylic residues in the undissociated form, the dialyzed solution was eluted through a DOWEX 50WX4-50 ion-exchange resin column previously treated with 2.0 M HCl. After freeze-drying, the new polymer was characterized by FT-IR and ^1^H-NMR spectra. FT-IR spectra were recorded with a Perkin Elmer Paragon 1000 spectrophotometer (USA) in the range 4,000-400 cm^-1^ using KBr pellets (number of scans 100, resolution of 1 cm^-1^). ^1^H-NMR spectra were obtained with a Bruker AC-400 instrument (Bruker, Germany) following a procedure already reported in the literature for other (1→3)-β-glucans [18]. In order to determine the number of carboxylic groups introduced on the scleroglucan, samples of the derivative (100 mg) were dissolved in water and submitted to potentiometric titration with 1x10^-2^ M NaOH. The amount of acid in mmol (equal to the mmol of employed base) was inserted into the equation:

amount of polymer (mg) = X PM_repetitive unit_ + (mmol of acid) PM_acid group_

and allowed the calculation of X, the mmol of repetitive units of the polymer. The ratio between the mmol of acid and the mmol of the repetitive units of the polymer multiplied by 100 gave the degree of derivatization (DD, number of carboxylic groups for 100 repetitive units) that was 65±5.

### 3.3. Studies of interaction with mucin

Since Scl and Scl-CM do not produce clear solutions in distilled water, the polymers were centrifuged (15 min, 8,000 rpm, 20 °C) in order to remove macro-aggregates eventually present in the solutions. Then the supernatant was recovered, lyophilized and the solid used for the evaluation of mucoadhesiveness. Ten mg of mucin were dispersed in a 0.1M phosphate buffered solution (8 mL, Na_2_HPO_4_, KH_2_PO_4_, pH 6.8) at 37 °C for 24 h under stirring. Then Scl, Scl-CM or hyaluronic acid (HA, chosen as a positive control) (10 mg) in pH 6.8 phosphate buffer (2 mL) were added to the mucin suspension (mucin/polymer weight ratio equal to 1) and each sample was incubated at 37 °C under stirring for 30 min, 2, 4, 6, 8 and 24 h. After each incubation time, the transmittance of the samples was recorded at 500 nm. Turbidity of the polymer-mucin mixtures was compared with that of a mucin dispersion containing the same mucin amount as the mixtures.

### 3.4. Hydrogel preparation

To a solution of Scl-CM (0.100 g in 5 mL of water, Cp= 2% w/v) maintained at 60 °C under stirring, different amounts of CaCl_2_ x 2H_2_O were added, in order to obtain salt concentrations (Cs) of 0.05, 0.10, 0.25 and 0.50 M, respectively. After complete dissolution, the solutions were left to cool to room temperature. The hydrogels containing the drugs were prepared dissolving the polymer (0.100 g, Cp= 2% w/v) in a solution of the drug (0.50 % w/v for NSAIDs, 0.25% for acyclovir) maintained at 60 ºC. After complete dissolution, CaCl_2_ x 2H_2_O was added in order to obtain Cs = 0.05 and 0.10 M respectively.

### 3.5. Release studies

Release experiments were carried out on samples (2.5 g) of freshly prepared hydrogels (Cp=2% w/v and Cs=0.05 and 0.10 M) containing the drug (0.50% w/v), with the rotating basket technique at 37.0 ± 0.1 °C and 100 rpm according to U.S.P. XXIV. The experiments were carried out with a SOTAX AT7 Smart (Switzerland) in 0.1M phosphate buffer (PB, pH=7.4) as release medium (0.5 L). The final concentration of the drug was always lower than 10% of its solubility in water [19], so that sink conditions could be assumed. The gel was carefully inserted into the basket and the drug release was followed by means of HPLC analysis, monitoring the amount of acyclovir at 255 nm, ibuprofen at 215 nm, ketoprofen and diclofenac at 253 nm. HPLC apparatus consisted of a Perkin Elmer Series 200 LC pump, equipped with a 235 Diode Array (USA). HPLC analyses were carried out using a Merck Hibar LiChrocart (250-4, 5 μm) RP-18 column, with a flow of 1 mL/min. CH_3_CN/H_3_PO_4_ 10^-2^ M mixture (7:3) was used as eluant for acyclovir and ibuprofen, CH_3_CN/H_3_PO_4_ 10^-2^ M mixture (1:1) for ketoprofen and diclofenac. All the experiments were carried out in triplicate and the results are reported as mean value ± SD.

### 3.6. Preparation of a double-layered system 

Fifty mg of scleroglucan were dissolved at 60 °C in 0.10 M CaCl_2_ (2.5 mL) containing 0.5% w/v of diclofenac sodium salt. The warm mixture was rapidly poured into a small glass (diameter=19 mm). Anther 50 mg of scleroglucan were dissolved at 60 °C in 0.05 M CaCl_2_ (2.5 m) containing 0.5% of diclofenac sodium salt and the warm gel was poured carefully on the first layer already cold. The release studies of diclofenac were carried out according to the method described before and using the same dissolution apparatus equipped with paddles instead of baskets, putting the glass with the double-layer system at the bottom of the vessel filled with 0.5 L of PB. 

### 3.7. Rheological measurements

Rheological experiments were performed with a Haake RheoStress 300 Rotational Rheometer (Germany) equipped with a Haake DC10 thermostat. Oscillatory experiments were performed at 25.0 ± 0.2 °C in the range 0.01-10 Hz on the hydrogels obtained adding a solution of CaCl_2_ to 2% w/v Scl-CM solutions in order to obtain final salt concentrations 0.05, 0.10, 0.25 and 0.50 M (Cp2-Cs0.05; Cp2-Cs0.1; Cp2-Cs0.25; Cp2-Cs0.5). Enough quantity of each sample was carefully poured to completely cover the 6 cm cone-plate geometry (angle of 1°). For each sample the linear viscoelastic range was evaluated: a 1% maximum deformation was used. Analogous experiments were carried out on the hydrogels loaded with diclofenac sodium salt.

### 3.8. In vivo studies 

Three healthy male New Zealand White rabbits were purchased from Charles River (Calco, Lecco, Italy) and acclimated to the laboratory for a week. The rabbits were individually housed and received standard diet and tap water ad libitum. Principles of laboratory animal care (EEC Directive of 1986; 86/609/EEC) guidelines were followed.

The back of the animals was clipped free of fur with an electric clipper 24 h before application of the sample. For the experiment, the clipped areas of skin of each rabbit were divided into four sites with the same area (30×30 mm). Scl, Scl-CM and the hydrogel Scl-CM with 0.1M CaCl_2_ were applied to three sites (approximately 500 mg/site); the last site was used as a control (no material present). The treated and the control sites were covered by gauze and the back of the rabbit was wrapped with a non-occlusive bandage, thereafter the animals were returned to their cages. After 4 h, the bandage and the test material were removed and 1 h later the sites were examined for skin irritation. Observation of the sites with material and control was repeated after 24, 48 and 72 h. The reaction, defined as erythema (Er) or edema (Ed), was evaluated according to the score of the skin reactions already reported [20].

The Score of Primary Irritation (SPI) was calculated for each rabbit as the difference between the sum of the scores for erythema (Er) and edema (Ed), at 24, 48 and 72 h divided by the number of the observations for the treated sites and the sum of the scores for erythema (Er) and edema (Ed), at 24, 48 and 72 h divided by the number of the observations for the control sites, according to the formula:
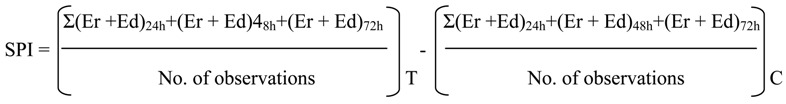
where T, treated; C, control; Er, erythema; Ed, edema. The PII was calculated as the arithmetical mean of the SPI values of the three animals. The evaluation of PII was performed according to the categories reported in the literature [20].

## 4. Conclusions

An easy to prepare and very versatile drug delivery system was obtained from an anionic derivative of scleroglucan having carboxylic moieties. This polymer is able to form hydrogels in the presence of Ca^+2^. The mechanical properties of the hydrogel, as well as its ability to control the release rate of an entrapped drug, critically depends on the Scl-CM/CaCl_2_ ratio. Therefore it was observed that the amount of the released drug could be easily modulated changing the salt concentration. Moreover by an opportune combination of hydrogels prepared using different salt concentrations, it was also possible to obtain a system with zero-order release kinetics. The rheological characteristics as well as the absence of skin irritation, confirm the potential of these matrices for the development of topical delivery systems.

## Figures and Tables

**Table 1:** Percentages of acyclovir released from Scl-CM based hydrogels with different salt concentration in 6 and 24 hours (C_p_=polymer concentration; C_s_=salt concentration). The results were reported as mean value ± SD.

Sample	Drug released in 6 h (%)	Drug released in 24 h (%)
C_p_2-C_s_0.50	16.2 ± 1.9	21.5 ± 1.2
C_p_2-C_s_0.25	40.2 ± 2.5	63.6 ± 0.5
C_p_2-C_s_0.10	90.6 ± 1.2	98.5 ± 1.2
C_p_2-C_s_0.05	92.7 ± 0.5	99.0 ± 0.3
